# The Attend Study: A Retrospective Observational Study of Emergency Department Attendances During the Early Stages of the COVID-19 Pandemic

**DOI:** 10.7759/cureus.9328

**Published:** 2020-07-21

**Authors:** Shu Hui Leow, Will Dean, Meiling MacDonald-Nethercott, Eoin MacDonald-Nethercott, Adrian A Boyle

**Affiliations:** 1 Emergency Medicine, University of Cambridge, School of Clinical Medicine, Cambridge, GBR; 2 Emergency Medicine, Addenbrookes Hospital, Cambridge University Hospitals Foundation Trust, Cambridge, GBR

**Keywords:** emergency department, epidemic, pandemic, ed attendances, ed crowding, covid-19, coronavirus, sars-cov-2, attendance

## Abstract

Introduction: The coronavirus disease-19 (COVID-19) pandemic was associated with a large reduction in the number of attendances at emergency departments (EDs) in March 2020 in the United Kingdom (UK). We sought to identify which patient groups attended EDs least.

Methods: Single-centre before and after study. We used routine administrative data from March 2020 and compared this to a composite control of March 2019 and February 2020.

Results: Mean daily attendance fell by 30% from 342 patients per day in the composite control months to 242 patients per day in March 2020. Reductions in attendance were seen in almost all patient groups but were greatest in patients with injuries, those referred by another clinician, those arriving at the weekend, and in patients who received no investigations. Multivariate analysis revealed that the proportion of patients who were admitted to hospital fell, despite the patients being sicker, older, needing more investigations, and more likely to arrive by ambulance.

Discussion: The reduction in ED attendances seen in the early phases of the UK pandemic occurred in all patient groups, but was greatest in the lower acuity patients. Reasons for this are complex and likely to be multifactorial.

## Introduction

The severe acute respiratory syndrome coronavirus 2 (SARS-CoV-2) virus was first detected in the United Kingdom (UK) on January 30, 2020 and has subsequently spread to cause over two hundred thousand confirmed infections [1]. NHS England data revealed a 25% fall in attendances to UK emergency departments (EDs) during March compared to recent months and previous years [2]. This follows over a decade of steadily increasing ED attendances and worsening ED crowding, despite efforts to reduce ED input to mitigate crowding [3-4]. However, the reduction in ED attendances is accompanied by decreases in diagnoses of nearly every condition, except pneumonias, sparking concerns that patients needing emergency care were not attending the ED [5].

The effect of viral epidemics on UK ED attendance has not been well studied but there are examples from recent epidemics elsewhere, such as during the severe acute respiratory syndrome (SARS) epidemic in 2003. In Singapore, there was an initial increase in ED attendances followed by a fall in numbers, seemingly corresponding to reports of intra-hospital spread of the SARS virus [6]. An ED in Taiwan recorded a 50% reduction in total attendances, with paediatric presentations 80% lower than the corresponding month in the previous year [7]. A fall in trauma and minor injury cases was also seen during another SARS outbreak in Hong Kong [8]. Similar findings were reported during a Middle Eastern respiratory syndrome (MERS) outbreak in Korea in 2015 - when it was noted that of the remaining presentations an increased proportion were of higher triage severity, but that the proportion of admissions remained at a similar level [9]. Conversely, a group of EDs in the US reported an increase in total attendances during the early stages of the H1N1 influenza outbreak in 2010 [10]. We had experienced a dramatic drop in the number of attendances arriving at our ED. The aim of this study was to describe the epidemiology of patients attending a single ED during the early stage of the pandemic, as this would provide useful insights about which patient groups are susceptible to behavioural interventions to reduce ED use.

## Materials and methods

Study design and setting

This is a retrospective observational cohort study comparing the demographic, attendance characteristics, and disposition of patients attending a Type 1 ED in Cambridge, UK, before and during the start of the COVID-19 outbreak in the UK. This ED sees around 120,000 attendances a year of all ages and has an admission rate of around 30% to observation and in-patient specialty wards. The hospital is entirely paperless and uses a single electronic patient record for all clinical encounters (Epic; Epic Systems Corporation, Verona, WI) and this provides a rich data set.

March 1-31, 2020 was selected as the intervention period as a convenience sample, and is likely to represent the beginning of the COVID-19 outbreak in the UK, with the first COVID-19 death reported on March 5, 2020 and a national lockdown announced on March 23, 2020. The control periods selected were those we thought to be the most similar to March 1, 2020 - February 29, 2020, and March 1-31, 2019. The mean of these two time periods were used for statistical analysis. There was little difference between the two control months. 

Data

Routine, depersonalised, administrative data on ED attendances were retrieved from the hospital’s electronic records system (Epic). All ED attendances were prospectively recorded by ED clinicians and ED administrative staff. Anonymised individual-level patient data were retrospectively extracted for all ED attendances during the study periods (n=28,882). Collected data fields were gender, age, arrival date and time, arrival mode, area of treatment, first National Early Warning Score 2 (NEWS2), referral source, attendance reason, disposal, and number of investigations whilst in the ED. 

Quantitative variables

Variables were chosen based on their ability to describe different categories of patients such as patient age, time of attendance, illness severity, other indicators of appropriateness of attendance, and quality of records of that specific variable. 

Multiple dichotomous variables were created for gender, time of attendance, NEWS2 score, and disposal description. Normal hours were defined as Monday to Friday, 9 am - 5 pm, and weekend attendances were defined as 00:00 Saturday - 23:59 Sunday. NEWS2 score was analyzed as >5 and >7, as it is used as an early warning score with two key trigger thresholds of 5 or more, and 7 or more [11]. The 21 disposal codes were condensed into two categories, admitted or discharged.

For the following, multiple categorical variables were created. Five treatment areas were identified as majors (trolley), resus, majors (ambulant), minors, and paeds. Twenty-five different referral sources were condensed into three categories - Ambulance, Registered Medical Professional, and Self-referral. Each attendance received between 0 - 18 investigations, which was condensed into three categories - those who received no investigations, those who received one investigation, and those who received two or more investigations, as clinicians are likely to order multiple investigations as part of a screening pack. 146 different attendance reasons were identified and condensed into eight categories - Abdominal, Cardac, Infectious, Injury, Neurological, Psychiatric, Respiratory, and Others. Multiple categorical variables were also created for age.

Statistical methods

The chi-squared test was used for categorical variables in the univariate analysis. We present unadjusted logistic regression, and also conducted a multivariable logistic regression analysis. All analyses were performed in STATA version 13 (StataCorp LP., College Station, TX). In the multivariate analysis we removed variables that we anticipated would have substantial collinearity, i.e. NEWS 2 of greater or equal to 7 and the area of the ED where people were being cared for. Where NEWS 2 was not recorded, we assigned this as zero, as this usually reflects patients with minor injuries or children.

Given that anonymised routinely collected data as part of a local service evaluation, no formal ethics review was sought. The HRA decision tool also identified this study as not needing formal ethics review. (http://www.hra-decisiontools.org.uk/ethics/EngresultN1.html) 

## Results

There were a total of 28,882 ED attendances during the study periods, of which 28,043 were included for analysis. 839 (2.9%) were excluded from analysis due to incomplete data records. Of these, 833 had missing information in one or more fields of arrival mode, treatment area, referral source or disposal description. One attendance was excluded with gender ‘N’, representing a patient who was not comfortable reporting their gender. Five attendances with unknown gender or age were also excluded. These represent an exceptional group of attendances that arrived at the ED unconscious or following major trauma, where it was impossible to collect information on gender or age. This group is extremely small and analysis would not yield meaningful results. 

During the months of March 2019 and February 2020 the number of mean (interquartile range) daily attendance was 342 (324-355). This reduced to 242 (176-321) during March 2020, a reduction of 30%. All patient groups reduced their attendance numbers, but the proportion of the reduction varied considerably.

Table 1 describes key demographics of the study groups. The gross reduction in daily attendances during March 2020 was seen across all patient age groups and gender but was greatest in people under 24 years of age. The biggest reduction in this time period was mean daily presentations as a result of injury (40%, 117 vs 73) whilst mean daily infection or respiratory related attendances fell the least, with a reduction of 10% (20 vs 18 and 32 vs 28 respectively). This relates to a 3.9% (34.2% vs 30.35% OR 0.94 CI 0.87-1.03) decrease in the proportion of presentations due to injury, and a 1.5% (5.8% vs 7.3% OR 1.34 CI 1.19 - 1.52) and 2.5% (9.3% vs 11.8% OR 1.35 CI 1.21 - 1.49) increase in infection or respiratory cases respectively. Statistically significant increases were seen in March 2020 in the proportion of ambulance arrivals (26.7% vs 31.4% +4.7% OR 1.26 CI 1.19-1.34) and proportion of patients with an initial NEWS 2 >5 (5% vs 5.9% +0.9% OR 1.18 CI 1.05-1.32), though the overall numbers were reduced. There was a reduction in the proportion of weekend attendances (30.4% vs 28.7% -1.7%, OR 0.92 CI 0.86-0.98). The unadjusted proportion of patients admitted from the ED did not change (33% baseline vs 32% study period OR 0.95 CI 0.9-1.01).

**Table 1:** Characteristics of Emergency Department Attendances in March 2020 Compared to Composite Control *p <0.05, **p <0.01

		Mar 2019 & Feb 2020		Mar 2020				
Average Daily Attendances		342		242				
		Daily Average	Proportion (%)	Daily Average	Proportion (%)	OR	p	95% CI
Female		173	51	123	51	0.99	0.75	0.94 - 1.05
Age								
	0-4	33	10	22	9	-	-	-
	5-15	33	10	21	9	0.97	0.66	0.86 - 1.10
	16-24	46	13	29	12	0.97	0.62	0.87 - 1.09
	25-34	45	13	34	14	1.15*	0.02	1.03 - 1.28
	35-44	34	10	26	11	1.17*	0.01	1.04 - 1.32
	45-54	34	10	25	10	1.14*	0.03	1.01 - 1.29
	55-64	31	9	23	9	1.12	0.06	0.99 - 1.27
	65-74	30	9	22	9	1.09	0.18	0.96 - 1.23
	75+	55	16	40	17	1.11	0.05	1.00 - 1.24
Arrival at the weekend		347	30	216	29	0.92*	0.01	0.87 - 0.98
Arrival by ambulance		91	27	76	31	1.26**	<0.01	1.19 - 1.34
‘NEWS 2' of 5 or more		17	5	14	6	1.18*	0.01	1.05 - 1.32
Referral Source								
	Registered clinical professional	78	23	46	19	-	-	-
	Self-referral	184	54	128	53	1.19**	<0.01	1.11 - 1.27
	Ambulance	80	23	68	28	1.44**	<0.01	1.33 - 1.56
Reason for Attendance								
	Abdominal	56	16	37	15	-	-	-
	Cardiac	29	8	22	9	1.16*	0.01	1.04 - 1.29
	Infectious	20	6	18	7	1.34**	<0.01	1.19 - 1.52
	Injury	117	34	73	30	0.94	0.17	0.87 - 1.03
	Neurological	58	17	40	17	1.05	0.32	0.96 - 1.15
	Psychiatry	10	3	8	3	1.18*	0.04	1.01 - 1.39
	Respiratory	32	9	28	12	1.35**	<0.01	1.21 - 1.49
	Other	21	6	15	6	1.09	0.17	0.96 - 1.24
Number of investigations								
	None	88	26	57	24	-	-	-
	One investigation	73	21	49	20	1.02	0.60	0.94 - 1.11
	More than one investigation	182	53	136	56	1.15**	<0.01	1.08 - 1.23
Admitted to hospital		113	33	77	32	0.95	0.10	0.90 - 1.01

**Figure 1:**
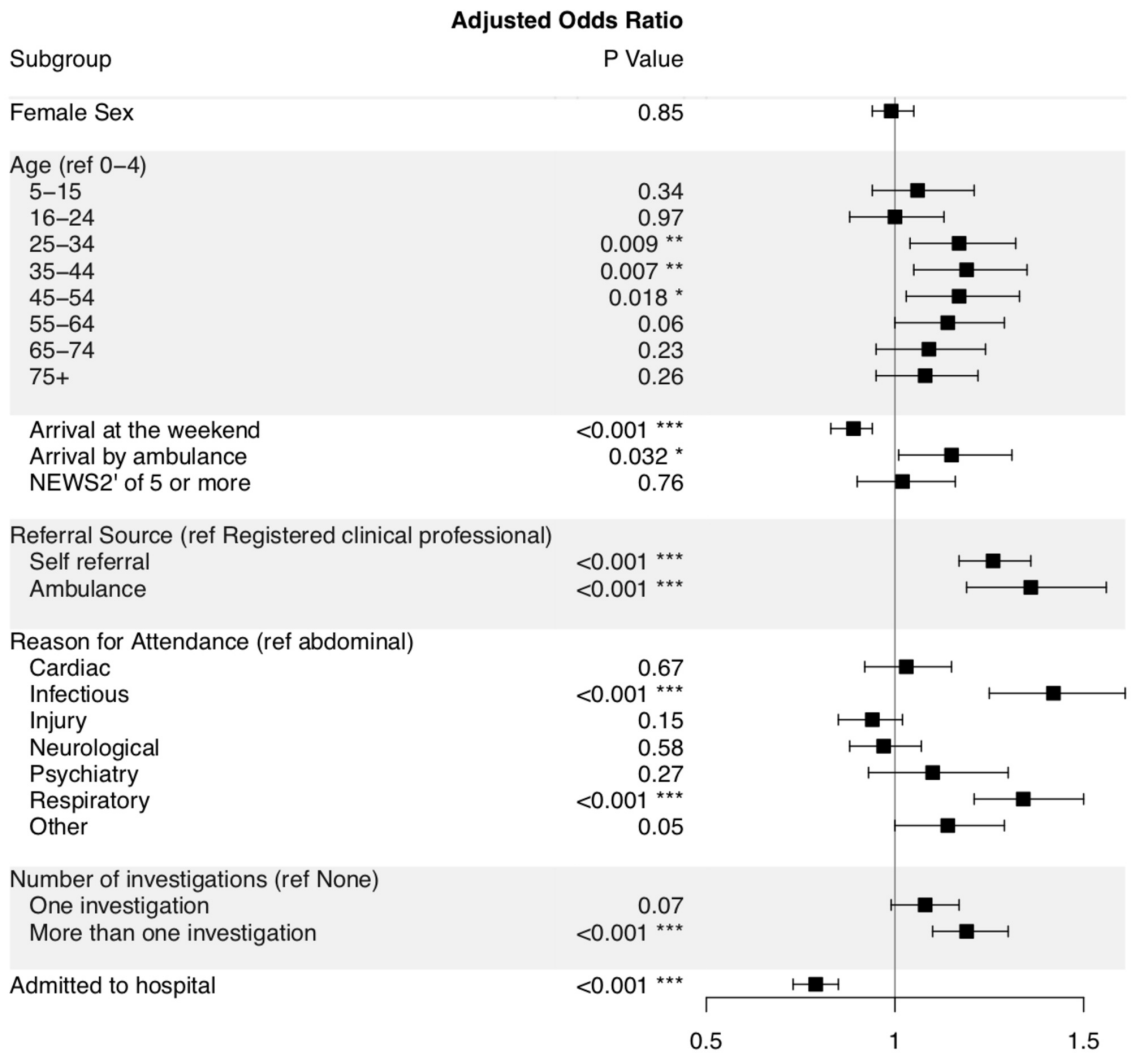
Forest Plot for Adjusted Odds Ratios of Emergency Department Attendances in March 2020 Compared to Composite Control * p<0.05, ** p<0.01, *** p<0.001

A multivariate analysis demonstrated that patients attending the ED in March 2020 were more likely to attend as a result of self-referral (OR 1.16 CI 1.17-1.36) or ambulance advice/transfer (OR 1.36 CI 1.19-1.56) (Table 2) (Figure 1). The gender and distribution remained unaffected after adjustment. There remains an increased proportion of respiratory (OR 1.34 CI 1.21-1.50) and infectious disease (OR 1.42 CI 1.25-1.61) presentations. There was no difference seen in the proportion of patients with an initial NEWS 2 >5 (OR 1.02 CI 0.9-1.16), but patients were more likely to have received more than one investigation (OR 1.19 CI 0.88-1.07). Within this adjusted analysis there was a large reduction in the likelihood of patients being admitted to the hospital from the ED (OR 0.79 CI 0.73 - 0.85). Goodness of fit was assessed with a Hosmer-Lemeshow test and was acceptable, Chi(df (8)=13.18, p=0.10).

**Table 2:** Multivariate Analysis of Emergency Department Casemix in March 2020 Compared to Composite Control *p <0.05, **p<0.01, ***p<0.001

			OR	p	95% CI
Female Sex			0.99	0.849	0.94 - 1.05
Age					
	0-4		REF	-	
	5-15		1.06	0.344	0.94 - 1.21
	16-24		1.00	0.971	0.88 - 1.13
	25-34		1.17**	0.009	1.04 - 1.32
	35-44		1.19**	0.007	1.05 - 1.35
	45-54		1.17*	0.018	1.03 - 1.33
	55-64		1.14	0.058	1.00 - 1.29
	65-74		1.09	0.225	0.95 - 1.24
	75+		1.08	0.257	0.95 - 1.22
Arrival at the weekend			0.89***	<0.001	0.93 - 0.94
Arrival by ambulance			1.15*	0.032	1.01 - 1.31
‘NEWS 2’ of 5 or more			1.02	0.757	0.90 - 1.16
Referral source					
	Registered clinical professional		REF	-	-
	Self-referral		1.26***	<0.001	1.17 - 1.36
	Ambulance		1.36***	<0.001	1.19 - 1.56
Reason for attendance					
	Abdominal		REF	-	
	Cardiac		1.03	0.667	0.92 - 1.15
	Infectious		1.42***	<0.001	1.25 - 1.61
	Injury		0.94	0.153	0.85 - 1.02
	Neurological		0.97	0.575	0.88 - 1.07
	Psychiatry		1.10	0.270	0.93 - 1.30
	Respiratory		1.34***	<0.001	1.21 - 1.50
	Other		1.14	0.050	1.00 - 1.29
Number of investigations					
		None	REF	-	-
		One investigation	1.08	0.073	0.99 - 1.17
		More than one investigation	1.19***	<0.001	1.10 - 1.30
Admitted to hospital			0.79***	<0.001	<0.001

## Discussion

During this period, the number of ED attendances decreased overall. This effect is seen most predominantly in younger patients, weekend attendances, and those presenting with injuries. The increase in proportion of infections and respiratory disease presentations may be attributed to COVID-19-related concerns, however, this was not proportionate. We noted a decrease in the proportion of patients presenting after a referral from healthcare professionals with a corresponding increase seen in both ambulance and self-referrals. Patients arriving to the department were more likely to require more than one investigation, but were not found to have an increased rate of initial NEWS 2 greater than 5. A key finding was that despite a similar unadjusted rate of admissions the likelihood of discharge from the ED was actually increased in the multivariable model.

There are some important limitations to this study. This was a single-centre study, however, the reduction in attendances was similar to the national picture. Variables chosen for analysis were further limited by the completeness and quality of routinely collected data. For instance, coding by clinicians is rarely completed accurately enough to allow analysis without substantial bias. In addition, we do not use, nor reliably collect and record, a formal triage system. However, this data source was sufficient for key demographics to be gathered and the amount of incomplete information on the final dataset was minimal. The detail that we were able to analyse is probably greater than for almost any other ED in the UK because of our Electronic Patient Record, where all clinical data is stored and recorded.

We speculate that the cause of the reduction in attendances to the ED is multifactorial. There are likely direct impacts from the social distancing measures and lockdown procedures that will lead to reduced sporting injuries, work related injuries, major trauma, and some alcohol related presentations. In addition, there may have been a ‘nosocomial dividend’ from reduced transmission of respiratory infections among small children. There may have been a reduction in the ‘worried well’ or those attending the ED with a self-limiting disorder, though the reduction in General Practitioner (GP) referrals was greater. However, the reduction in all age groups and presenting complaints appears to also represent a reduced level of concerning pathology. Indeed both the US and Italy have reported reduced incidence of acute coronary syndrome [12-13]. Our data cannot answer if there has been a true reduction in the incidence of disease or whether patients are avoiding attending ED and cases are contributing to excess community deaths. 

Reduction in proportion of referrals from a registered clinical professional may be due to reduced NHS services including specialist clinics and community health services [14], and patients assuming their GP may not be available. Alternatively, as per a reduction in hospital admissions, it may represent GPs finding alternative routes to treat patients and avoiding secondary care. This could represent an avenue for further investigation to continue to minimise ED crowding during the ongoing COVID-19 epidemic and post-COVID-19 plans.

The striking result from the multivariate analysis was that after correction for other variables, the adjusted rate of hospital admissions was significantly reduced. This may be the result of more low risk patients being discharged, for instance, plastic surgery or orthopedic teams not admitting patients awaiting theatre the next day. Equally there may have been a reduction in admissions at the expense of prolonged time in the ED whilst teams waited for further results. However, more optimistically this could be a positive effect due to early senior review as speciality rotas were being covered by consultant staff. There may also have been an increased risk threshold for admission altering clinician/patient behaviour, and improved shared decision making about avoiding acute hospital admissions. The reduced attendance numbers may have reduced crowding in the department and allowed earlier review and subsequent discharge, as crowding has been previously associated with increased admissions of low acuity cases [3]. 

Our work has some important policy implications. Firstly, performance standards during this time such as the four-hour access standard cannot be meaningfully compared, as our data shows that case mix has dramatically altered. Secondly, the reduction in referrals from registered clinicians, who are overwhelmingly GPs, suggests that this area of admission avoidance can be effective, though it is not clear what the demand on GPs was during this time.

## Conclusions

Our findings describe a large, unprecedented decrease in daily ED attendances during the start of the COVID-19 pandemic in UK compared to the preceding month and the same month the previous year, consistent with the national trend. Analysis of patient data demonstrated changes in ED casemix with reduced levels of concerning pathology, in line with findings from Italy and the US, although it is unclear whether this represents a true decrease in incidence of disease. A multivariate analysis also found changes in admissions and referral levels, that may have arisen from changes in behaviour of healthcare professionals and accessibility of healthcare services in response to the COVID-19 pandemic. Finally, the findings of this study may guide future interventions to reduce ED crowding and indicates that the usual service evaluation standards for EDs may not be comparable during this time.

## References

[1] England PH (2020). Coronavirus (COVID-19) cases in the UK. https://www.gov.uk/government/publications/covid-19-track-coronavirus-cases.

[2] Digital N (2020). NHS Digital: Hospital Episode Statistics (HES). https://digital.nhs.uk/data-and-information/data-tools-and-services/data-services/hospital-episode-statistics.

[3] Morris ZS, Boyle A, Beniuk K, Robinson S (2012). Emergency department crowding: towards an agenda for evidence-based intervention. Emerg Med J.

[4] Powis S (2020). NHS England: clinically-led review of NHS access standards - interim report from the NHS National Medical Director. https://www.england.nhs.uk/wp-content/uploads/2019/03/CRS-Interim-Report.pdf.

[5] Thornton J (2020). Covid- 19: A&E visits in England fall by 25% in week after lockdown. BMJ.

[6] Lateef F (2004). SARS changes the ED paradigm. Am J Emerg Med.

[7] Huang H-H, Yen DH-T, Kao W-F, Wang L-M, Huang C-I, Lee C-H (2006). Declining emergency department visits and costs during the severe acute respiratory syndrome (SARS) outbreak. J Formos Med Assoc.

[8] Man CY, Yeung RSD, Chung JYM, Cameron PA (2003). Impact of SARS on an emergency department in Hong Kong. Emerg Med Australas.

[9] Paek SH, Kim DK, Lee JH, Kwak YH (2017). The impact of Middle East respiratory syndrome outbreak on trends in emergency department utilization patterns. J Korean Med Sci.

[10] McDonnell WM, Nelson DS, Schunk JE (2012). Should we fear “flu fear” itself? Effects of H1N1 influenza fear on ED use. Am J Emerg Med.

[11] Royal College of Physicians (2020). Royal College of Physicians: National Early Warning Score (NEWS) 2: standardising the assessment of acute-illness severity in the NHS. National Early Warning Score (NEWS) 2: Standardising the assessment of acute-illness severity in the NHS. Updated report of a working party.

[12] Solomon MD, McNulty EJ, Rana JS (2020). The Covid-19 pandemic and the incidence of acute myocardial infarction [Epub ahead of print]. N Engl J Med.

[13] De Filippo O, D’Ascenzo F, Angelini F (2020). Reduced rate of hospital admissions for ACS during Covid-19 outbreak in northern Italy. N Engl J Med.

[14] Winn M, Hayter A (2020). NHS England: COVID-19 prioritisation within community health services. https://www.england.nhs.uk/coronavirus/wp-content/uploads/sites/52/2020/03/C0145-COVID-19-prioritisation-within-community-health-services-1-April-2020.pdf.

